# Effect of pre-stroke statin use on stroke severity and early functional recovery: a retrospective cohort study

**DOI:** 10.1186/s12883-015-0376-3

**Published:** 2015-07-30

**Authors:** Jay Chol Choi, Ji Sung Lee, Tai Hwan Park, Yong-Jin Cho, Jong-Moo Park, Kyusik Kang, Kyung Bok Lee, Soo-Joo Lee, Youngchai Ko, Jun Lee, Joon-Tae Kim, Kyung-Ho Yu, Byung-Chul Lee, Jae-Kwan Cha, Dae-Hyun Kim, Juneyoung Lee, Dong-Eog Kim, Myung Suk Jang, Beom Joon Kim, Moon-Ku Han, Hee-Joon Bae, Keun-Sik Hong

**Affiliations:** Department of Neurology, Jeju National University Hospital, Jeju National University, Jeju, South Korea; Clinical Research Center, Asan Medical Center, Seoul, South Korea; Department of Neurology, Seoul Medical Center, Seoul, South Korea; Department of Neurology, Ilsan Paik Hospital, Inje University, Goyang, South Korea; Department of Neurology, Eulji General Hospital, Eulji University, Seoul, South Korea; Department of Neurology, Soonchunhyang University College of Medicine, Seoul, South Korea; Department of Neurology, Eulji University Hospital, Daejeon, South Korea; Department of Neurology, Yeungnam University Hospital, Daegu, South Korea; Department of Neurology, Chonnam National University Hospital, Gwangju, South Korea; Department of Neurology, Hallym University Sacred Heart Hospital, Anyang, South Korea; Department of Neurology, Dong-A University College of Medicine, Busan, South Korea; Department of Biostatistics, Korea University College of Medicine, Seoul, South Korea; Department of Neurology, Dongguk University Ilsan Hospital, Goyang, South Korea; Department of Neurology, Seoul National University Bundang Hospital, Seoul National University College of Medicine, Seongnam, South Korea

**Keywords:** Acute stroke, Statins, Outcomes

## Abstract

**Background:**

Experimental studies suggest that pre-stroke statin treatment has a dual effect of neuroprotection during ischemia and neurorestoration after ischemic injury. The aim of this study was to evaluate the effect of pre-stroke statin use on initial stroke severity and early clinical outcome.

**Methods:**

We used a prospective database enrolling patients with acute ischemic stroke from 12 hospitals in Korea between April 2008 and January 2012. Primary endpoint was the initial stroke severity as measured by the National Institutes of Health Stroke Scale (NIHSS) score. Secondary endpoints were good outcome (modified Rankin Scale [mRS], 0–2) and overall mRS distribution at discharge. Multivariable regression model and propensity score (PS) matching were used for statistical analyses.

**Results:**

Among the 8340 patients included in this study, 964 patients (11.6 %) were pre-stroke statin users. The initial NIHSS score (mean [95 % CI]) was lower among pre-stroke statin users vs. non-users in multivariable analysis (5.7 [5.2–6.3] versus 6.4 [5.9–6.9], *p* = 0.002) and PS analysis (5.2 [4.7–5.7] versus 5.7 [5.4–6.0], *p* = 0.043). Pre-stroke statin use was associated with increased achievement of mRS 0–2 outcome (multivariable analysis: OR [95 % CI], 1.55 [1.25–1.92], *p* < 0.001; PS matching: OR [95 % CI], 1.47 [1.16-1.88]; *p* = 0.002) and favorable shift on the overall mRS distribution (multivariable analysis: OR [95 % CI], 1.29 [1.12-1.51], *p* = 0.001; PS matching: OR [95 % CI], 1.31 [1.11-1.54]; *p* = 0.001).

**Conclusions:**

Pre-stroke statin use was independently associated with lesser stroke severity at presentation and better early functional recovery in patients with acute ischemic stroke.

**Electronic supplementary material:**

The online version of this article (doi:10.1186/s12883-015-0376-3) contains supplementary material, which is available to authorized users.

## Background

Statin use before cerebral ischemia was associated with a smaller infarction volume or more collaterals in patients with an acute large cerebral artery occlusion [1, 2], which are in accord with experimental statin studies showing a neuroprotective effect during ischemia and a neurorestorative effect after ischemic injury [3–9]. However, the effects of pre-stroke statin use on clinical stroke severity and functional outcomes have been inconsistent [2, 10–24]. Pre-stroke statin use was independently associated with milder stroke severity in limited studies [15, 21]. Improved functional outcomes in patients with pre-stroke statin use have been demonstrated in multiple studies [10–12, 15, 16, 18, 19, 24], but it is not clear whether the improved functional outcome was attributed to a neuroprotective effect leading to milder stroke severity at presentation or to a neurorestorative effect after ischemia. Small sample sizes, selection bias, or limited availability of detailed information in human clinical studies are likely to result in these conflicting results. We tested a hypothesis that whether pre-stroke statin use is associated with initial stroke severity as well as early stroke recovery, by analyzing a large dataset from a multicenter registry that prospectively and consecutively enrolled patients with acute ischemic stroke.

## Methods

### Database and subjects

We used data from the Clinical Research Center for Stroke-5 (CRCS-5) registry, which is a prospective registry of consecutive patients with acute ischemic stroke or transient ischemic attack (TIA) admitted to 12 academic centers in Korea (http://www.stroke-crc.or.kr). The CRCS-5 registry was launched in April 2008 to facilitate multicenter collaborative clinical stroke research in Korea and to implement clinical practice guidelines for stroke [25], and was approved by the Institutional Review Boards (IRBs) of all participating centers (Jeju National University Hospital, Seoul Medical Center, Ilsan Paik Hospital, Eulji General Hospital, Soonchunhyang University Hospital, Eulji University Hospital, Yeungnam University Hospital, Chonnam National University Hospital, Hallym University Sacred Heart Hospital, Dong-A University, Seoul National University Bundang Hospital, and Dongguk University Ilsan Hospital). The informed consent from individual patients or their legally authorized representatives was waived by the relevant IRBs because the registry aimed to monitor and improve the quality of stroke care and a computer-assisted de-identification system ensured the anonymity of individual patients during data collection. The Steering Committee of the CRCS-5 Registry approved the access and analysis of the database.

For this study, we analyzed the CRCS-5 dataset of patients hospitalized with acute ischemic stroke between April 1, 2008 and January 31, 2012. Inclusion criteria were (1) age ≥18 years, and (2) arrival at the emergency room (ER) within 48 h from symptom onset. Exclusion criteria were (1) pre-stroke disability as measured by a modified Rankin Scale (mRS) score of >1; (2) unavailability of discharge mRS; (3) TIA patients without relevant neuroimaging findings; (4) unavailability of ischemic stroke subtype of Trial of Org 10172 in Acute Stroke Treatment (TOAST) classification; and (5) patients who were treated with thrombolytic therapy. For patients treated with thrombolytic therapy, the success of thrombolytic therapy would predominantly affect the early post-stroke functional outcome. Therefore, we excluded those patients from the primary analysis cohort. Data of patients treated with thrombolytic therapy and all patients, including those treated with and not treated with thrombolytic therapy, were additionally analyzed and presented in the supporting information.

### Data collection

Using a web-based registry which provided a pre-defined standardized coding system, we prospectively and systematically captured the following data for each patient: (1) demographics of age, sex, height, weight, body mass index (kg/m^2^), and systolic and diastolic blood pressure at admission; (2) laboratory findings of glucose at admission, fasting total cholesterol, and fasting LDL cholesterol; (3) vascular risk factors of hypertension, diabetes mellitus, hyperlipidemia, smoking, atrial fibrillation, and prior history of stroke and coronary artery disease; (4) use of medications prior to the index stroke including antiplatelet agents, medications for hypertension, diabetes mellitus, and hyperlipidemia including statins; and (5) characteristics of the index stroke including pre-stroke and discharge functional disability measured by mRS score, initial stroke severity measured by the National Institutes of Health Stroke Scale (NIHSS) score, and use of thrombolytic therapy. Ischemic stroke was classified as large-artery atherosclerosis (LAA), small-vessel occlusion (SVO), cardioembolism (CE), stroke of undetermined etiology (UDE) or stroke of other determined etiology (ODE) according to the TOAST criteria [26]. Symptomatic stenosis or occlusion (SYSO) of the major arteries was assessed using computed tomography angiography, magnetic resonance angiography, or conventional catheter angiography. For intracranial arteries, it was defined as more than 50 % stenosis of the artery compared with proximal or distal normal segment [27]. In this study, pre-stroke statin use was defined as taking statin at the time of the index ischemic stroke. Participating investigators and research nurses were trained and obtained certification for the NIHSS and mRS scoring using a web-based certification program provided in the CRCS website (http://www.stroke-crc.or.kr).

### Outcome measurement

The primary outcome was the initial stroke severity as measured by the NIHSS score. The secondary outcome was early post-stroke functional outcomes as assessed by mRS score at discharge.

### Statistical analysis

For missing data with more than 10 % of total observation was excluded from the analysis. We used simple imputation with median value for variables with missing data less than 10 % of total observation. Baseline characteristics between pre-stroke statin users and non-users were compared with Student *t*-test for continuous variables with a normal distribution, Wilcoxon rank-sum test for continuous variables without a normal distribution, or the chi-square test for categorical variables. When comparing the primary endpoint of the initial NIHSS score between the two groups, the Wilcoxon rank-sum test was used for a univariable analysis and an analysis of covariance (ANCOVA) test was used to adjust for covariates, which showed a p-value <0.25 on the comparisons of baseline characteristics between the two groups. Additionally, we compared the proportion of mild stroke (ie, NIHSS score 0 to 4) between two groups as a dichotomized outcome. To further explore a differential effect of prior statin use on initial stroke severity by ischemic stroke subtypes, we compared the initial NIHSS scores between the two groups stratified by the TOAST classification. For the stratified analysis, we combined UDE and ODE subtypes. Multivariable analyses according to individual TOAST subtypes included covariates that were selected for the adjusted analysis of the primary endpoint. However, the variable of the TOAST subtype was excluded.

When analyzing mRS scores at discharge, we compared the proportion of mRS scores of 0–2 and the overall distribution of mRS scores between the two groups. For the analysis of the overall mRS distribution, we employed 6 levels by collapsing mRS 5 and mRS 6 into a single level of extreme disability or death [28]. The odds ratio (OR) and 95 % confidence interval (CI) were calculated to estimate the probabilities of achieving a discharge mRS 0–2 outcome and a favorable shifting of one level on the mRS score for statin users. To adjust covariates, multiple logistic regression was conducted for the dichotomized mRS outcome and ordinal logistic regression analysis for the overall mRS distribution. Covariates were adjusted in the same way as for the primary endpoint, and the initial NIHSS score and statin use during hospitalization were additionally included. We used the following formula to calculate number needed to treat (NNT) using OR and control event rate (CER) [29].

$$ \mathrm{N}\mathrm{N}\mathrm{T}=\left[\mathrm{C}\mathrm{E}\mathrm{R}\left(\mathrm{OR}-1\right)+1\left]/\right[\mathrm{C}\mathrm{E}\mathrm{R}\left(\mathrm{OR}-1\right)\times \left(1-\mathrm{C}\mathrm{E}\mathrm{R}\right)\right] $$

Because neurological status of stroke patients tends to change during acute phase, we performed stratified analysis by median onset to arrival time to investigate whether there is a difference of prestroke statin effect according to onset to arrival time.

In addition to multivariable analyses, we conducted propensity score (PS) analyses to reduce the bias due to confounding variables. To obtain PS, we used logistic regression, in which pre-stroke statin use was employed as a dependent variable. The model included all patient pretreatment characteristics with respect to prior statin use. After obtaining PS, statin users were 1-to-n (n ranged from one to four) matched to non-users within 0.2 × SD of the logit of the propensity score. Standardized differences of covariates were used to assess baseline imbalances between the two groups after PS matching. Using the final PS matched dataset, outcome analysis was performed with the generalized estimation equation method. For covariates with more than 0.1 standardized differences were further adjusted on the matched analysis. In addition, stratification by deciles of the PS was used as a sensitivity analysis. In all analyses, a p-value <0.05 was considered statistically significant. SAS computer software (Version9.3, SAS Institute, Cary, NC, USA) was used for the statistical analyses.

## Results

### Study population

Of the 14,746 patients with acute ischemic stroke or TIA enrolled in the CRCS-5 registry between April 1, 2008 and January 31, 2012, we excluded 6406 patients in the following order: (1) 12 patients <18 years old; (2) 3310 patients arriving at the ER beyond 48 h from symptom onset; (3) 1255 patients with a pre-stroke mRS score > 1; (4) 15 patients without documentation of discharge mRS score; (5) 170 patients of TIA without relevant acute ischemic lesions on neuroimaging; (6) 68 patients without documentation of the TOAST classification; and (7) 1576 patients treated with intravenous or intra-arterial thrombolysis (Additional file 1: Figure S1).

The study population of the current analysis included 8340 patients. The mean age was 66.8 years (standard deviation [SD], 12.7), and 59.6 % were men. The median length of hospitalization was 8 days (interquartile range [IQR], 6–13). The median NIHSS score at presentation was 3 (IQR, 2–7). LAA (36.4 %) was the most common ischemic stroke subtype, followed by SVO (23.5 %) and CE (20.5 %). Other baseline characteristics are presented in Table 1. Missing values were found on seven variables, which ranged from 0.1 % to 2.2 % of the total observations (Additional file 1: Table S1).

**Table 1:** Demographic and clinical characteristics of statin users and non-users

	Before PS matching			After PS matching		
	Statin users (*n* = 964)	Non-users (*n* = 7376)	*P*-value*	Statin users (*n* = 618)	Non-users (*n* = 1585)	*P*-value**
Demographic						
Mean age (SD), years	68.3 (10.6)	66.6 (12.9)	<0.001	67.9 (10.9)	67.4 (11.9)	0.91
Male sex, *n*(%)	539 (55.9)	4433 (60.1)	0.013	345 (55.8)	891 (56.2)	0.99
Mean BMI (SD), kg/m^2^	24.1 (3.3)	23.6 (3.7)	<0.001	23.9 (3.3)	23.8 (3.4)	0.99
Pre-stroke mRS, *n*(%)			<0.001			0.68
0	834 (86.5)	6909 (93.7)		552 (89.3)	1458 (92.0)	
1	130 (13.5)	467 (6.3)		66 (10.7)	127 (8.0)	
Risk factors, *n*(%)						
Hypertension	800 (83.0)	4765 (64.6)	<0.001	482 (78.0)	1200 (75.7)	0.89
DM	458 (47.5)	2250 (30.5)	<0.001	269 (43.5)	643 (40.6)	0.90
Hyperlipidemia	807 (83.7)	1771 (24.0)	<0.001	461 (74.6)	1007 (63.5)	0.74
Smoking	344 (35.7)	3068 (41.6)	<0.001	219 (35.4)	575 (36.3)	0.92
Atrial fibrillation	210 (21.8)	1307 (17.7)	0.002	133 (21.5)	305 (19.2)	0.76
History of stroke	345 (35.8)	1153 (15.6)	<0.001	186 (30.1)	400 (25.2)	0.80
History of CAD	147 (15.3)	272 (3.7)	<0.001	58 (9.4)	135 (8.5)	0.59
Lab, mean(SD)						
SBP, mmHg	148.5 (26.9)	149.2 (27.4)	0.46	148.6 (26.1)	149.4 (28.0)	0.89
DBP, mmHg	84.2 (15.0)	87.0 (15.6)	<0.001	85.5 (15.0)	85.8 (16.3)	0.98
Hemoglobin	13.4 (1.9)	13.8 (1.9)	<0.001	13.6 (1.9)	13.6 (1.9)	0.56
Admission glucose, mg/dL	124.5 (51.1)	123.4 (52.4)	0.38	126.7 (51.9)	126.6 (52.8)	0.81
Total cholesterol, mg/dL	164.0 (42.0)	185.3 (40.8)	<0.001	173.8 (43.3)	180.1 (42.6)	0.67
LDL cholesterol, mg/dL	93.6 (33.3)	113.4 (35.4)	<0.001	101.6 (34.8)	106.8 (37.3)	0.67
Stroke characteristics						
Median onset to arrival time (IQR), hours	7.0 (2.5 - 19.5)	7.7 (3.0 - 20.0)	0.040	7.1 (2.5 - 19.9)	7.4 (2.8 - 19.8)	0.99
Stroke subtype, *n*(%)						0.98
Large artery atherosclerosis	361 (37.5)	2678 (36.3)	<0.001	226 (36.6)	597 (37.7)	
Small vessel occlusion	168 (17.4)	1790 (24.3)	<0.001	116 (18.8)	334 (21.1)	
Cardioembolism	234 (24.3)	1477 (20.0)	<0.001	153 (24.8)	352 (22.2)	
Others	201 (20.9)	1431 (19.4)	<0.001	123 (19.9)	302 (19.1)	
SYSO	409 (42.4)	3011 (40.8)	0.34	257 (41.6)	651 (41.1)	0.81
Pre-stroke medication, *n*(%)						
Antiplatelet	671 (69.6)	1479 (20.1)	<0.001	351 (56.8)	722 (45.6)	0.92
Anticoagulant	85 (8.8)	251 (3.4)	<0.001	45 (7.3)	99 (6.2)	0.93
ARB or ACEI	435 (45.1)	1221 (16.6)	<0.001	225 (36.4)	484 (30.5)	0.60
Beta-blocker	207 (21.5)	485 (6.6)	<0.001	104 (16.8)	221 (13.9)	0.93
Diuretics	179 (18.6)	554 (7.5)	<0.001	93 (15.0)	209 (13.2)	0.86
Calcium channel blocker	327 (33.9)	1124 (15.2)	<0.001	170 (27.5)	404 (25.5)	0.85

*SD* Standard deviation, *PS* Propensity score, *BMI* Body mass index, *mRS* modified Rankin scale, *DM* Diabetes mellitus, *CAD* Coronary artery disease, *SBP* Systolic blood pressure, *DBP* Diastolic blood pressure, *IQR* Interquartile range, *SYSO*, Symptomatic stenosis or occlusion, *ARB* Angiotensin-receptor blocker, *ACEI* Angiotensin converting enzyme inhibitor

*P-values are calculated by Student's *t*-test, Pearson chi-square test, or Wilcoxon rank sum test as appropriate

**P-values are calculated by conditional logistic regression

Of the 8340 patients, 964 (11.6 %) were taking statins at the time of the index stroke onset. Compared with non-users, pre-stroke statin users were more likely to be older and women, to have a pre-stroke disability of mRS 1, a history of hypertension, diabetes mellitus, hyperlipidemia, atrial fibrillation, coronary artery disease, and prior stroke, and to have already been on antiplatelet agents, angiotensin-receptor blockers (ARBs) or angiotensin converting enzyme inhibitors (ACEIs), beta-blockers, diuretics, or calcium channel blockers (CCBs). However, they had lower fasting total and LDL cholesterol levels. The distribution of TOAST classification also differed between the two groups. After PS matching, the baseline characteristics did not differ significantly between the pre-stroke statin users and non-users (Table 1). During hospitalization, 6258 patients (75.0 %) received statin treatment, and 123 patients (12.8 %) of 964 pre-stroke statin users did not receive statins. The reasons for the statin withdrawal were not documented.

For patients treated with thrombolytic therapy and all patients including those treated with and not treated with thrombolytic therapy, the baseline imbalances between pre-stroke statin users and non-users were generally similar to those of patients not treated with thrombolytic therapy (Additional file 1: Tables S2 and S3).

### Primary outcome

**Fig. 1:**
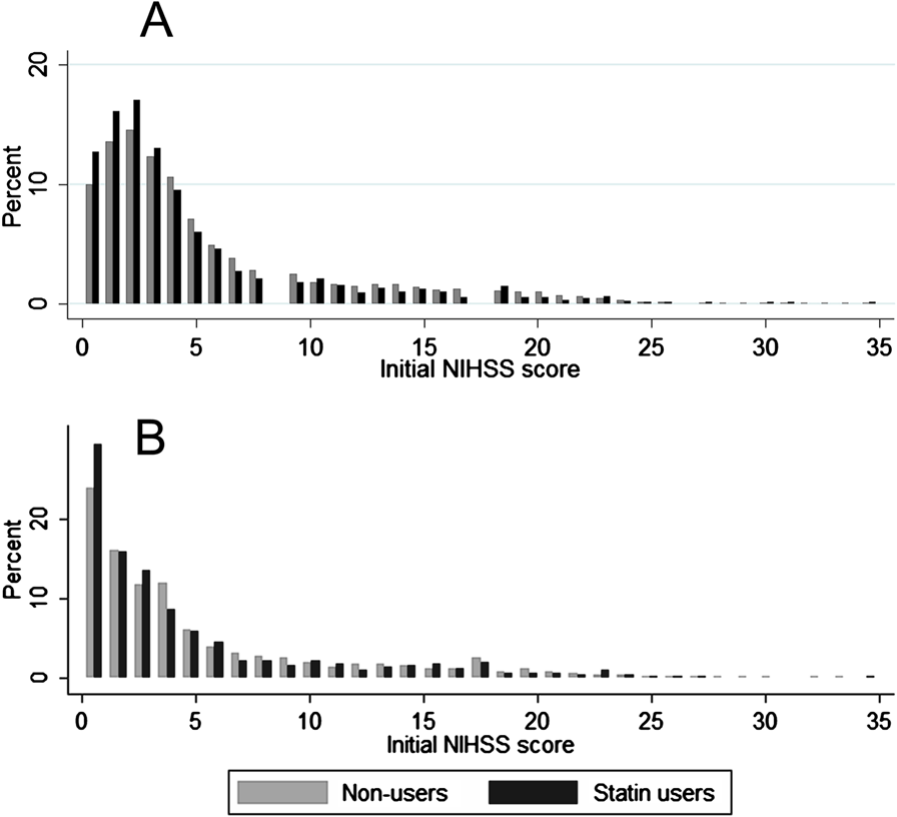
Histogram showing initial NIHSS scores in statin users and non-users of unmatched (*n* = 8340) (**a**) and PS-matched cohort (*n* = 2203) (**b**)

The mean initial NIHSS score (95 % CI) was 4.6 (4.3–4.9) among pre-stroke statin users and 5.4 (5.3–5.6) among non-users. Accordingly, prior statin use was associated with an average decrease of 0.8 points on the initial NIHSS score (95 % CI, 0.5–1.2; *p* < 0.001) (Table 2). The median initial NIHSS score did not differ numerically between the two groups, but the difference was statistically significant because of the difference in the NIHSS score distributions: 3 (IQR, 1–6) in pre-stroke statin users versus 3 (IQR, 2–7) in non-users (unadjusted analysis, *p* < 0.001) (Fig. 1A). After adjusting for age, sex, body mass index, diastolic blood pressure, hemoglobin, total cholesterol, LDL cholesterol, pre-stroke mRS, hypertension, diabetes mellitus, hyperlipidemia, atrial fibrillation, history of prior stroke and coronary heart disease, smoking, prior use of an antiplatelet medication, ARB or ACEI, beta-blocker, diuretic, CCB, onset-to-arrival, and TOAST classification, the mean initial NIHSS score remained significantly lower in statin users than in non-users (5.7 [5.2–6.3] versus 6.4 [5.9–6.9]; ANCOVA test, p = 0.002) (Table 2).

**Table 2:** Comparisons of initial NIHSS scores between statin users and non-users for unmatched and PS-matched cohorts

	Statin users	Non-users	Difference	*P*-value
Unmatched cohort				
Unadjusted	4.6 (4.3–4.9)	5.4 (5.3–5.6)	0.8 (0.5–1.2)	<0.001
Adjusted^a^	5.7 (5.2–6.3)	6.4 (5.9–6.9)	0.7 (0.2–1.1)	0.002
PS-matched cohort				
PS-matched^b^	5.2 (4.7–5.7)	5.7 (5.4–6.0)	0.5 (0.02–1.0)	0.043
PS-stratification, deciles^c^	5.1 (4.7–5.6)	5.7 (5.6–5.9)	0.6 (0.1–1.1)	0.015

Values are mean (95 % CI) or least-square mean (95 % CI) as appropriate

^a^Adjusted for age, sex, body mass index, diastolic blood pressure, hemoglobin, total cholesterol, LDL cholesterol, pre-stroke modified Rankin Scale score, history of hypertension, diabetes mellitus, hyperlipidemia, atrial fibrillation, history of stroke, history of coronary artery disease, smoking, prior antiplatelet medication, anticoagulant, angiotensin receptor blocker or angiotensin converting enzyme inhibitor, beta-blocker, diuretics, calcium-channel blocker, TOAST classification, SYSO and onset to arrival time

^b^PS-matched sample included 618 pairs with one-to-n (n ranged from one to four) matching: 618 statin users and 1585 non-users. Adjusted for hyperlipidemia, history of stroke, total cholesterol, LDL cholesterol, prior medications of any antiplatelet and ARB or ACEI, and SYSO

^c^Adjusted for SYSO

*NIHSS* National Institutes of Health Stroke Scale, *PS* Propensity score

Values presented are type III estimates

PS-matched sample included 619 pairs with 619 statin users and 1585 non-users. The estimated logistic regression model and PS model equations were provided in the online supplemental material (Additional file 1: Table S4). Covariates including history of stroke, total cholesterol, LDL cholesterol, and prior medications of any antiplatelet and ARB or ACEI were further adjusted on PS-matching analysis since they had more than 0.1 standardized differences between statin user and non-users after the PS matching (Additional file 1: Figure S2). The difference in the initial NIHSS scores between pre-stroke statin users and non-users remained significant in both the PS-matched cohort (5.2 [4.7–5.7] versus 5.7 [5.4–6.0], *p* = 0.043) (Fig. 1B) and the PS-stratification cohort (5.1 [4.7–5.6] versus 5.7 [5.6–5.9], *p* = 0.015) (Table 2). The adjusted analysis using all patients data showed that pre-stroke statin users compared to non-users were more likely to present as mild stroke defined as NIHSS score 0–4. However, after PS matching, the findings were not significant, but the direction favored pre-stroke statin use (Additional file 1: Table S4).

Among TOAST subtypes, univariable analyses showed that prior statin use was associated with lower initial NIHSS scores in patients with LAA, SVO, and UDE or ODE, but not in those with CE (Additional file 1: Figure S3). After adjusting for covariates, the significance disappeared for all TOAST subtypes, but in patients with LAA and SVO, there were trends of lower initial NIHSS scores among pre-stroke statin users (Additional file 1: Table S5).

When analyzing the 1576 patients who were treated with thrombolytic therapy, the initial NIHSS score did not differ between statin users and non-users (12.0 [6.5–17.0] vs 12.0 [6.0–17.0], unadjusted analysis) (Additional file 1: Table S2). For 9916 patients, including non-thrombolysed and thrombolysed patients, the initial NIHSS score was lower in statin users than in non-statin users (unadjusted mean [95 % CI], 5.9 [5.5–6.3] vs 6.5 [6.3–6.6], *p* < 0.001; adjusted mean 7.2 [6.6–7.7] vs 7.6 [7.2–8.1], *p* = 0.038). Differences in the initial NIHSS score by TOAST classification showed a similar pattern as observed in patients who were not treated with thrombolytic therapy. (Additional file 1: Table S6).

### Secondary outcome

**Fig. 2:**
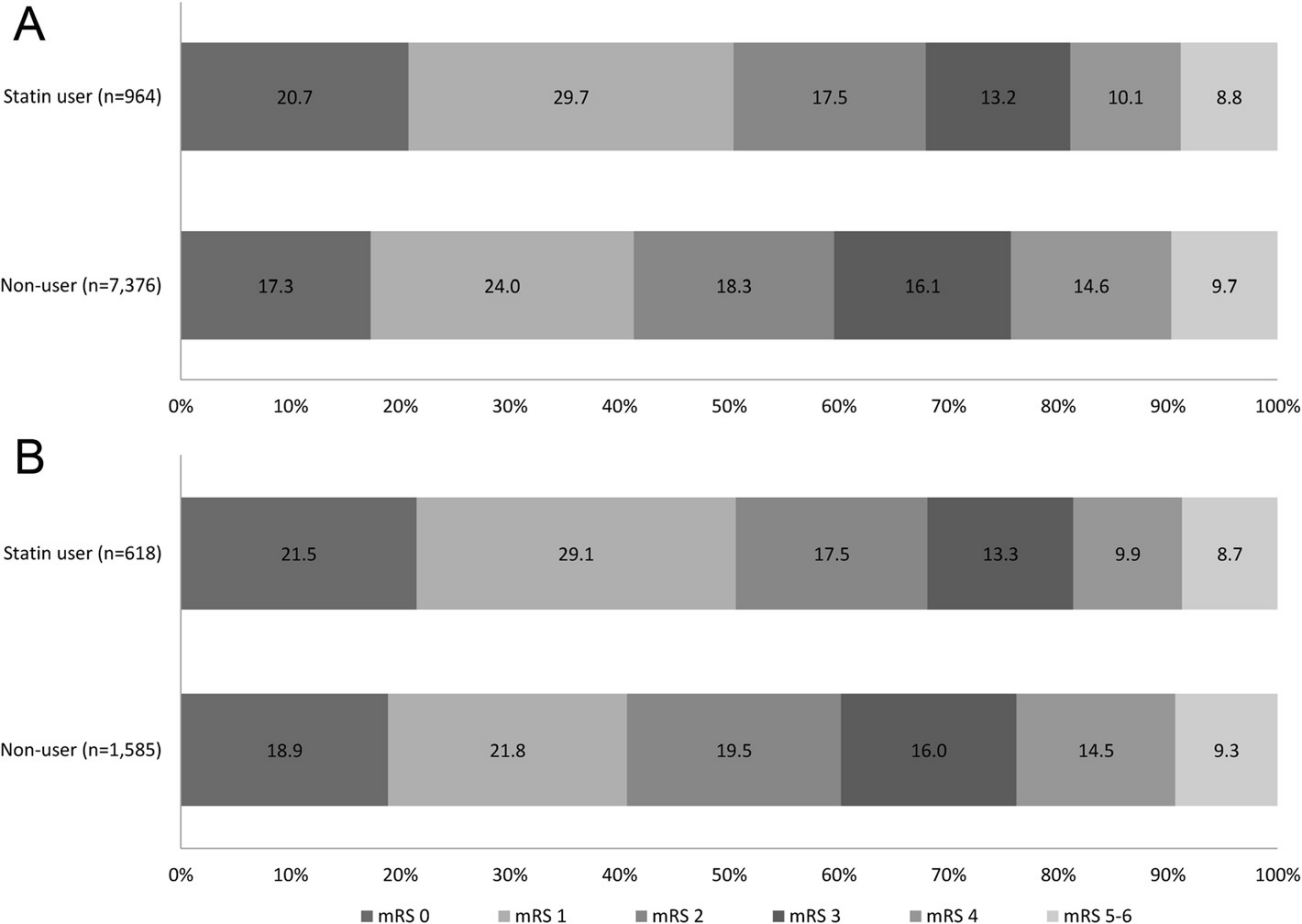
Distribution of modified Rankin Scale (mRS) score at discharge (**a**. unmatched, **b**. PS-matched)

A good functional outcome of mRS 0–2 at discharge was achieved in 655 patients (68.0 %) among pre-stroke statin users and 4395 patients (59.6 %) among non-users, representing an absolute difference of 8.4 % (unadjusted OR, 1.44; 95%CI, 1.24–1.66; *p* < 0.001) and an NNT of 11.9. The difference remained significant after adjusting for covariates including the initial NIHSS score and statin use during hospitalization (adjusted OR, 1.55; 95 % CI, 1.25–1.92; *p* < 0.001) (Table 3). Fig. 2 shows the distribution of discharge mRS outcomes of the two groups before and after PS analysis. In an unadjusted analysis, pre-stroke statin use favorably shifted the distribution of mRS outcomes (Cochran-Mantel-Haenszel test, *p* < 0.001). After adjusting for covariates, the association of statin use with the favorable shift in the mRS outcomes remained significant (adjusted OR, 1.30; 95%CI, 1.12–1.51; *p* = 0.001) (Table 3).

**Table 3:** Multivariable binary and ordinal logistic regression analyses for mRS outcome for unmatched cohort

	Binary logistic regression^a^			Ordinal logistic regression^b^		
	OR	95 % CI	P-value	OR	95 % CI	P-value
Demographic						
Age, years	0.98	(0.97–0.98)	<0.001	0.98	(0.98–0.99)	<0.001
Sex, male	1.20	(1.04–1.38)	0.012	1.08	(0.97–1.20)	0.157
Pre-stroke mRS						
1	Ref		Ref	Ref		
0	1.31	(1.06–1.63)	0.012	1.54	(1.31–1.81)	<0.001
BMI	1.00	(0.99–1.02)	0.65	1.00	(0.99–1.01)	0.64
Risk factors						
Hypertension	1.03	(0.91–1.18)	0.63	1.01	(0.92–1.11)	0.87
DM	0.79	(0.70–0.88)	0 < .001	0.79	(0.73–0.86)	<0.001
Hyperlipidemia	0.86	(0.75–0.98)	0.024	0.94	(0.85–1.04)	0.20
Smoking	0.93	(0.82–1.07)	0.32	1.04	(0.94–1.14)	0.48
Atrial fibrillation	1.27	(1.01–1.61)	0.046	1.11	(0.94–1.14)	0.24
History of stroke	0.99	(0.85–1.16)	0.90	0.97	(0.86–1.09)	0.58
History of CAD	0.79	(0.61–1.02)	0.068	0.88	(0.73–1.06)	0.178
Lab						
DBP, (unit 10 mmHg)	0.94	(0.91–0.98)	0.002	0.95	(0.92–0.97)	<0.001
Hemoglobin	1.04	(1.01–1.08)	0.023	1.03	(1.01–1.06)	0.015
Total cholesterol, (unit 10 mg/ dL)	0.95	(0.93–0.98)	<0.001	0.96	(0.94–0.98)	<0.001
LDL cholesterol, (unit 10 mg/ dL)	1.05	(1.01–1.08)	0.005	1.03	(1.00–1.05)	0.022
Stroke characteristics						
Initial NIHSS score	0.76	(0.74–0.77)	<0.001	0.76	(0.75–0.76)	<0.001
Onset to arrival time	1.00	(0.99–1.00)	0.0719	0.99	(0.99–1.00)	<0.001
Stroke subtype						
Large artery atherosclerosis	Ref		Ref	Ref		
Small vessel occlusion	1.10	(0.87–1.39)	0.42	1.06	(0.90–1.26)	0.50
Cardioembolism	1.42	(1.21–1.67)	<0.001	1.13	(1.01–1.27)	0.035
Others	1.14	(0.97–1.33)	0.120	1.08	(0.96–1.21)	0.20
Pre-stroke medication						
Antiplatelet	0.97	(0.87–1.13)	0.72	0.95	(085–1.06)	0.35
Anticoagulant	0.98	(0.72–1.33)	0.888	1.03	(0.83–1.27)	0.81
ARB or ACEI	1.11	(0.95–1.29)	0.19	1.02	(0.92–1.15)	0.67
Beta-blocker	1.40	(1.12–1.75)	0.003	1.26	(1.08–1.48)	0.003
Diuretics	1.02	(0.83–1.25)	0.86	0.97	(0.84–1.13)	0.73
Calcium channel blocker	0.92	(0.79–1.08)	0.31	1.02	(0.91–1.15)	0.71
Statin use during hospitalization	1.09	(0.95–1.25)	0.22	1.26	(1.14–1.39)	<0.001
SYSO	0.81	(0.71–0.91)	0.001	0.81	(0.74–0.89)	<0.001
Pre-stroke statin use	1.55	(1.25–1.92)	<0.001	1.30	(1.12–1.51)	0.001

*mRS* Modified Rankin scale, *BMI* Body mass index, *DM* Diabetes mellitus, *DBP* Diastolic blood pressure, *CAD* Coronary artery disease, *NIHSS* National Institutes of Health Stroke Scale, *ARB* Angiotensin-receptor blocker, *ACEI* Angiotensin converting enzyme inhibitor, *SYSO* Symptomatic stenosis or occlusion

^a^Dependent variable: mRS 0 to 2 versus 3 to 6

^b^Dependent variable: six levels by collapsing mRS 5 and mRS 6 into a single level

To examine the effect of statin initiation during hospitalization among patients without pre-stroke statin use, we compared discharge mRS between statin users (*n* = 5428) and statin non-users (*n* = 1892) during hospitalization. In unadjusted analyses, statin initiation during hospitalization was associated with better discharge mRS outcome in both binary and ordinal analyses. After adjusting covariates including initial NIHSS score, statin initiation was not associated with better mRS outcome in binary analysis (OR, 1.08; 95 % CI, 0.94–1.24; *p* = 0.31), but the association was significant in ordinal analysis (OR, 1.26; 95 % CI, 1.14–1.40; *p* < 0.001) (Additional file 1: Table S7).

In the PS-matched cohort, pre-stroke statin use was associated with favorable mRS outcome in both binary (adjusted OR [95 % CI], 1.47 [1.16–1.88]; *p* = 0.002) and ordinal analyses (adjusted OR [95 % CI], 1.26 [1.06–1.50]; *p* = 0.008). In the PS-stratification cohort, which served as sensitivity analysis, pre-stroke statin use was also significantly associated with good functional outcome as well as a favorable shift in the mRS outcomes (Table 4).

**Table 4:** Multivariable binary and ordinal logistic regression analyses for mRS outcome for unmatched and PS-matched cohorts

	Binary outcome^a^			Ordinal outcome^b^		
	OR	95 % CI	p-value	OR	95 % CI	p-value
Crude analysis, unmatched cohort	1.44	(1.25–1.66)	<0.001	1.37	(1.21–1.54)	<0.001
Multivariable analysis^c^, unmatched cohort	1.55	(1.25–1.92)	<0.001	1.29	(1.12–1.51)	0.001
PS- matched analysis^d, e^	1.47	(1.16–1.88)	0.002	1.26	(1.06–1.50)	0.008
PS-stratification, deciles^f^	1.57	(1.25–1.96)	<0.001	1.31	(1.11–1.54)	0.001

Odd ratio for statin use prior to stroke

^a^Dependent variable: mRS 0 to 2 versus 3 to 6

^b^Dependent variable: six mRS levels by collapsing mRS 5 and mRS 6 into a single level

^c^Adjusted for age, sex, body mass index, diastolic blood pressure, hemoglobin, total cholesterol, LDL cholesterol, pre-stroke modified Rankin scale score, history of hypertension, diabetes mellitus, hyperlipidemia, atrial fibrillation, history of stroke, history of coronary artery disease, smoking, prior medication of any antiplatelet, anticoagulant, angiotensin receptor blocker or angiotensin converting enzyme inhibitor, beta-blocker, diuretics, calcium-channel blocker, TOAST classification, onset to arrival time, statin use during hospitalization, SYSO, and initial NIHSS score

^d^PS-matched sample included 618 pairs with one-to-n (n ranged from one to four) matching: 618statin users and 1585 non-users

^e^Adjusted for history of stroke, hyperlipidemia, total cholesterol, LDL cholesterol, prior medication of any antiplatelet, ARB + ACEI, statin use during hospitalization, SYSO, and initial NIHSS score

^f^Adjusted for statin use during hospitalization, SYSO, and initial NIHSS score

Among stroke subtypes, multivariable analyses showed that the association of pre-stroke statin use with achieving a mRS 0–2 outcome at discharge was significant in patients with CE, whereas the association of pre-stroke statin use with a favorable shifting on the discharge mRS score was significant in those with LAA (Additional file 1: Table S8).

When analyzing data of patients treated with thrombolytic therapy, pre-stroke statin use was not associated with an improvement of discharge mRS outcomes on shift analysis (Cochran-Mantel-Haenszel test, *p* = 0.461) as well as on dichotomized analysis (unadjusted OR, 1.03; 95 % CI, 0.75–1.41; *p* = 0.857). For 9916 patients, including non-thrombolysed and thrombolysed patients, pre-stroke statin was associated with a good outcome of mRS 0–2 at discharge (adjusted OR, 1.41; 95%CI, 1.16–1.71; *p* = 0.0004) as well as a favorable shifting on the discharge mRS (adjusted OR, 1.22; 95%CI, 1.06–1.40; *p* = 0.0063) after adjustment for covariates (Additional file 1: Table S9).

To investigate the effect of prestroke statin by onset to arrival time, we performed stratified analysis using median onset to arrival (7.6 h). For initial stroke severity, the prestroke statin effect was significant in patients arriving within 7.6 h, but not in patients arriving after 7.6 h, and the interaction was significant (Additional file 1: Table S10). However, for functional outcome, there was no significant interaction for dichotomized mRS analyses and shift analyses. In the mRS 0–2 dichotomized analysis, the prestroke statin effect was significant irrespective of onset to arrival time. In the shift analysis, the effect was significant in patients arriving within 7.6 h, but showed a non-significant trend in those arriving after 7.6 h (Additional file 1: Table S11).

## Discussion

This study shows that pre-stroke statin use was associated with lesser stroke severity at presentation. The magnitude of the statin benefit on initial stroke severity was relatively small: a decrease of 0.8 points of the NIHSS score for the unadjusted analysis and a decrease of 0.6 points for the adjusted and PS analyses. However, the small decrease in the NIHSS score might be clinically meaningful because a 1-point decrease in the NIHSS score was significantly associated with reduction of hospitalization, the need for rehabilitation or a long-term nursing facility [30, 31].

Most of the earlier studies have failed to detect the benefit of pre-stroke statin use on initial stroke severity [2, 10–14, 16–20, 23]. However, except for two studies [16, 19], they had a small sample size of less than 1000 and accordingly were not adequately powered to detect the pre-stroke statin effect on initial stroke severity. Only two studies have demonstrated the beneficial effect of pre-stroke statin on initial stroke severity [15, 21]. Both studies showed that pre-stroke statin was independently associated with a higher probability of mild stroke severity as defined by the NIHSS score of 0–5, but they did not provide the pre-stroke statin effect on the overall stroke severity after adjusting for covariates. When comparing the NIHSS score between two groups, the analysis of the overall NIHSS score distribution is preferred to a dichotomized analysis because of 1) retaining all stroke severity information captured by the NIHSS score, 2) being free from a cut-off point bias which is frequently observed in dichotomized analyses, and 3) better detecting a therapeutic effect that is mild to modest. In addition, of the two studies, one study specifically analyzed the statin effect [21], but the other study assessed the effect of lipid-lowering therapy including statins and other lipid-lowering drugs [15]. In our study, the impact of pre-stroke statin on the overall stroke severity was more convincingly demonstrated by ensuring a substantial statistical power and enabling an extensive covariate adjustment as well as PS analyses.

In accord with earlier observational studies, we found that pre-stroke statin use was associated with good functional outcome at discharge. Compared to non-users, statin users had a 1.55-fold increased odds of achieving a good outcome (mRS 0–2) and a 1.29-fold increased odds of a favorable single level transition on the mRS disability score after adjustment for covariates including the initial NIHSS score and statin use during hospitalization. The internal validity of our findings is supported by the consistent magnitudes of the associations between multivariable analyses and PS analyses for both of the dichotomized and shift analyses (Table 4). The external validity is supported by the comparable magnitude of benefit reported in a recent meta-analysis that pooled 9 studies of 17,512 patients and assessed mRS 0–2 at discharge or at 30 days: the odds ratio (95 % CI) of pre-stroke statin use for achieving a mRS 0–2 outcome was 1.55 (1.25–1.92) in our study versus 1.64 (1.14–2.36) in the meta-analysis [22]. In addition, the favorable shift on the mRS score observed in the current study is generally comparable to the results of an exploratory analysis of the SPARCL trial, which analyzed the 90-day mRS outcome of 454 patients experiencing recurrent ischemic stroke during the trial (197 patients randomized to high dose atorvastatin versus 257 to placebo) [24]. In the absence of randomized trials specifically designed to test the effect of pre-stroke statin on stroke outcome, data from such randomized trial settings are likely to better ensure baseline balances of two comparative groups than observational studies. However, the SPARCL exploratory analysis was not adequately powered to detect a favorable mRS shift. In addition, that study was not able to adjust the initial stroke severity of the recurrent ischemic stroke because of the unavailability of the data. In contrast, our study with a larger sample size that assessed the initial NIHSS score for each patient was able to observe a favorable mRS shift and to adjust for the initial stroke severity.

Animal experiments have shown that pretreatment with statin have neuroprotective actions of enhancing angiogenesis, reduction of clot formation or facilitation of clot lysis, and upregulation of endothelial nitric oxide synthase [4–6, 9], and statin treatment after stroke had neurorestorative actions of promoting neurogenesis, synaptogenesis, and angiogenesis [8]. Thereby, pre-stroke statin use might affect not only initial stroke severity but also early stroke recovery. However, no prior human stroke study has demonstrated both of these effects simultaneously. Several studies showed better functional outcome but failed to show lesser initial stroke severity in patients with pre-stroke statin use [10–12, 16, 18, 19]. On the contrary, in one study, pre-stroke statin was associated with lesser stroke severity but not with better functional outcome [21]. In another study, pre-stroke lipid-lowering therapy of statin or fibrate was independently associated with both mild stroke severity and early good functional outcome. However, in an additional multivariable model including initial stroke severity, the association of pre-stroke lipid-lowering therapy and good functional outcome was not significant. Therefore, the better functional outcome might be attributed to the lesser stroke severity rather than early post-stroke recovery [15]. In contrast, we found that pre-stroke statin use was associated with both lesser stroke severity and better functional outcome even after adjusting for initial stroke severity, suggesting that pre-stroke statin in human strokes might lead to not only lesser stroke severity but also early stroke recovery.

Previous studies have shown that pretreatment with statin for statin-naïve patients or reloading of high dose statin reduced myocardial infarction in patients undergoing percutaneous coronary intervention for stable angina or acute coronary syndrome [32–34], and current guidelines state that high-dose statin therapy before percutaneous coronary intervention is reasonable [35, 36]. In contrast, stroke guidelines do not clearly state whether statins should be initiated immediately or delayed during the acute period of ischemic stroke. In a small randomized trial, statin withdrawal for a brief period of 3 days in acute ischemic stroke patients who were already taking statins was associated with increased risk of death or dependency at 3 months [37]. On the basis of the results, the current guidelines recommend that continuation of statin therapy during the acute period of ischemic stroke is reasonable for patients already taking statins at the time of ischemic stroke onset [38]. In this study, statin withdrawal among pre-stroke statin users had a trend of worse mRS outcome, but the association was not statistically significant. The insufficient statistical power due to a small sample size might in part account for the negative association. In contrast, among pre-stroke statin non-users, statin initiation during hospitalization was significantly associated with a favorable mRS shift. Our results along with other studies suggest that 1) for patients at high risk of cardiovascular disease, statins should be recommended to ameliorate the disability from brain or heart attack as well as to prevent these events; and 2) during the acute stage of ischemic stroke, continuation of statin therapy for patients on chronic statin therapy and immediate statin initiation for statin-naïve patients might be beneficial to improve functional outcome after stroke.

Statin effect on stroke severity and functional outcome might differ depending on ischemic stroke subtypes. In earlier studies, pre-stroke statin was not associated with mild stroke severity in all stroke subtypes [16], whereas it was associated with better functional outcome in strokes due to SVO and LAA [16, 17]. In the current study, for LAA and SVO, the association of pre-stroke statin with lesser stroke severity was significant in unadjusted analyses but showed trends without statistical significance in adjusted analyses. For early functional outcome, adjusted analyses showed more mRS 0–2 outcomes in CE and favorable mRS shifts in LAA with pre-stroke statin use. Limited statistical power due to small to modest sample sizes for individual stroke subtypes in the current and previous studies limits the interpretation of the inconsistent findings.

Our study has several limitations. This was an observational study, which could not control unmeasured confounders. In addition, data were prospectively collected in but retrospectively abstracted from a registry database and thereby might not be as accurate as those from clinical trials. However, to ensure the accuracy of data, we used a pre-defined standardized coding system and data quality was audited regularly. The effect on stroke outcome might differ across statin types, doses, and durations, as suggested in experimental studies [6, 39]. Since our registry did not capture relevant data, this study was not able to analyze these effects. Although a recent study did not find a dose-dependent effect of pre-stroke statin on initial stroke severity, the results might be attributed to lack of statistical power [21]. We assessed mRS outcomes at discharge, but could not analyze the 90-day mRS outcome disability, which is recommended as a preferred functional outcome measure in acute stroke research [40, 41]. However, an earlier study analyzing the NINDS-TPA (National Institute of Neurological Disorders and Stroke rt-PA) trial database showed that mRS at day 7/10 strongly correlated with the 90-day mRS [42]. Finally, we could not find a significant association between prestroke statin and functional outcome in patients treated with thrombolytic therapy due to small sample size. Future clinical trials should validate the effect of prestroke statin in patients with acute stroke.

## Conclusions

The present study suggests that pre-stroke statin use might be associated with milder stroke severity at presentation and better early recovery in patients with acute ischemic stroke. Our findings need to be replicated in other well-designed studies.

## Additional file

Additional file 1:
**Table S1**. Report on missing data (*n*=8340). **Table S2**. Baseline characteristics, initial NIHSS score, and discharge outcome of statin users and non-users in patients who received thrombolytic therapy (*n*=1576). **Table S3**. Baseline characteristics and discharge outcome of statin users and non-users in patients including non-thrombolysed and thrombolysed patients (*n*=9916). **Table S4**. Odd ratios for dichotomized NIHSS score by prestrroke statin use (*n*=8340). **Table S5**. Comparison of the initial NIHSS Scores between statin users and non-users in patients not treated with thrombolytic therapy (*n*=8340). **Table S6**. Comparison of the initial NIHSS Scores between statin users and non-users for all patients including non-thrombolysed and thrombolysed patients (*n*=9916). **Table S7**. Comparisons of discharge mRS outcome by statin use during hospitalization in pre-stroke statin non-users. **Table S8**. Adjusted odds ratios of achieving a mRS 0–2 outcome and favorable shifting of the mRS score among pre-stroke statin users stratified by TOAST subtypes (*n*=8340). **Table S9**. Multivariable analysis for favorable mRS outcome for all patients (*n*=9916). **Table S10**. Comparisons of initial NIHSS scores between statin users and non-users by median onset to arrival time. **Table S11**. Comparisons of discharge mRS outcomes by median onset to arrival time. **Figure S1**. Study flow diagram. **Figure S2**. Standardized difference of covariates before and after propensity score matching. **Figure S3**. Comparison of the initial NIHSS scores by ischemic stroke subtype in 8340 patients.

## Abbreviations

NIHSS: National Institutes of Health Stroke Scale
mRS: modified Rankin Scale, PS, propensity score
CRCS-5: Clinical Research Center for Stroke-5
TIA: Transient ischemic attack
IRBs: Institutional Review Boards
ER: Emergency room
TOAST: Trial of Org 10172 in Acute Stroke Treatment
LAA: Large-artery atherosclerosis
SVO: Small-vessel occlusion
CE: Cardioembolism
UDE: Stroke of undetermined etiology
ODE: Stroke of other determined etiology
SYSO: Symptomatic stenosis or occlusion
ANCOVA: An analysis of covariance
OR: Odds ratio
CI: Confidence interval
NNT: Number needed to treat
CER: Control event rate
ARB: Angiotensin-receptor blocker
ACEI: Angiotensin converting enzyme inhibitors
CCB: Calcium channel blockers
