# Supplementary material for: Effect of pre-stroke statin use on stroke severity and early functional recovery: a retrospective cohort study
Source: BMC Neurol. 2015 Jul 30;15:120. doi: 10.1186/s12883-015-0376-3 (PMC4520077; doi:10.1186/s12883-015-0376-3)
Supplement: Additional file 1: — Table S1. Report on missing data (n=8340). Table S2. Baseline characteristics, initial NIHSS score, and discharge outcome of statin users and non-users in patients who received thrombolytic therapy (n=1576). Table S3. Baseline characteristics and discharge outcome of statin users and non-users in patients including non-thrombolysed and thrombolysed patients (n=9916). Table S4. Odd ratios for dichotomized NIHSS score by prestrroke statin use (n=8340). Table S5. Comparison of the initial NIHSS Scores between statin users and non-users in patients not treated with thrombolytic therapy (n=8340). Table S6. Comparison of the initial NIHSS Scores between statin users and non-users for all patients including non-thrombolysed and thrombolysed patients (n=9916). Table S7. Comparisons of discharge mRS outcome by statin use during hospitalization in pre-stroke statin non-users. Table S8. Adjusted odds ratios of achieving a mRS 0–2 outcome and favorable shifting of the mRS score among pre-stroke statin users stratified by TOAST subtypes (n=8340). Table S9. Multivariable analysis for favorable mRS outcome for all patients (n=9916). Table S10. Comparisons of initial NIHSS scores between statin users and non-users by median onset to arrival time. Table S11. Comparisons of discharge mRS outcomes by median onset to arrival time. Figure S1. Study flow diagram. Figure S2. Standardized difference of covariates before and after propensity score matching. Figure S3. Comparison of the initial NIHSS scores by ischemic stroke subtype in 8340 patients. [file 12883_2015_376_MOESM1_ESM.pdf]

Additional Files

Table S1. Report on missing data (*n*=8,340)

Variable	System missing, *n*	User-defined missing, *n*	Out of range values, *n*	Total missing, *n*	%
Height	0	160	0	160	1.9%
Weight	0	152	0	152	1.8%
Total cholesterol	0	92	1	93	1.1%
LDL cholesterol	0	187	0	187	2.2%
Admission glucose	4	177	0	181	2.2%
Systolic blood pressure	0	5	0	5	0.1%
Diastolic blood pressure	0	5	0	5	0.1%

Table S2. Baseline characteristics, initial NIHSS score, and discharge outcome of statin users and non-users in patients who received thrombolytic therapy (*n*=1,576)

	Statin users (*n*=196)	Non-users (*n*=1,380)	P-value*
Demographic			
Mean age (SD), years	69.8 (9.8)	66.8 (12.8)	<0.001
Male sex,*n*(%)	114 (58.2)	826 (59.9)	0.65
Mean BMI (SD), kg/m^2	24.3 (3.1)	23.4 (3.2)	<0.001
Pre-stroke mRS, *n*(%)			0.001
0	171 (87.2)	1293 (93.7)	
1	25 (12.8)	87 (6.3)	
Risk factors, *n*(%)			
Hypertension	175 (89.3)	851 (61.7)	<0.001
Diabetes mellitus	72 (36.7)	328 (23.8)	<0.001
Hyperlipidemia	158 (80.6)	292 (21.2)	<0.001
Smoking	77 (39.3)	534 (38.7)	0.87
Atrial fibrillation	86 (43.9)	529 (38.3)	0.137
History of stroke	70 (35.7)	173 (12.5)	<0.001
History of CAD	45 (23.0)	66 (4.8)	<0.001
Lab, mean(SD)			
Systolic blood pressure, mmHg	151.6 (27.8)	147.3 (28.1)	0.047
Diastolic blood pressure, mmHg	84.9 (17.0)	86.8 (17.8)	0.145
Admission glucose, mg/dL	130.6 (57.7)	120.7 (43.6)	0.006
Total cholesterol, mg/dL	154.6 (38.8)	182.2 (40.1)	<0.001
LDL cholesterol, mg/dL	87.4 (33.0)	111.3 (34.6)	<0.001
Stroke characteristics			
Median onset to arrival time (IQR), hours	1.7 (0.6-1.8)	2.2 (0.6-2.3)	0.158
Mean initial NIHSS score (95%CI)	12.2 (11.3–13.2)	12.0 (11.7–12.4)	0.68
Median initial NIHSS score (IQR)	12 (6.5–17)	12 (6–17)	0.74
Stroke subtype, *n*(%)			0.34
LAA	56 (28.6)	421 (30.5)	
SVO	8 (4.1)	89 (6.5)	
CE	91 (46.4)	561 (40.7)	
UDE or ODE	41 (20.9)	309 (22.4)	
Discharge mRS 0 to 2	83 (42.4)	575 (41.7)	0.86
Pre-stroke medication, *n*(%)			
Any antiplatelet	132 (67.4)	301 (21.8)	<0.001
ARB+ACEI	85 (43.4)	203 (14.7)	<0.001
Beta-blocker	60 (30.6)	124 (9.0)	<0.001
Diuretic	40 (20.4)	113 (8.2)	<0.001
Calcium channel blocker	70 (35.7)	211 (15.3)	<0.001
SYSO	142 (72.5)	964 (69.9)	0.46

* P-values are calculated by Student's t-test, Pearson chi-square test, or Wilcoxon rank sum test, as appropriate

mRS, modified Rankin scale; CAD, coronary artery disease; ARB, angiotensin-receptor blocker; ACEI, angiotensin converting enzyme inhibitor; NIHSS, National Institutes of Health Stroke Scale; SD, standard deviation; IQR, interquartile range; LAA, large-artery atherosclerosis; SVO, small-vessel occlusion; CE, cardioembolism; UDE, stroke of undetermined etiology; ODE, stroke of other determined etiologies; SYSO, symptomatic stenosis or occlusion

Table S3. Baseline characteristics and discharge outcome of statin users and non-users in patients including non-thrombolysed and thrombolysed patients (*n*=9,916)

	Statin users (*n*=1,160)	Non-users (*n*=8,756)	P-value*
Demographic			
Mean age (SD), years	68.5 (10.5)	66.6 (12.9)	<0.001
Male sex,*n*(%)	653 (56.3)	5,259 (60.1)	0.014
Mean BMI (SD), kg/m^2	24.2 (3.3)	23.6 (3.7)	<0.001
Pre-stroke mRS, *n*(%)			<0.001
0	1,005 (86.6)	8,202 (93.7)	
1	155 (13.4)	554 (6.3)	
Risk factors, *n*(%)			
Hypertension	975 (84.1)	5,616 (64.1)	<0.001
Diabetes mellitus	530 (45.7)	2,578 (29.4)	<0.001
Hyperlipidemia	965 (83.2)	2,063 (23.6)	<0.001
Smoking	421 (36.3)	3,602 (41.1)	0.002
Atrial fibrillation	296 (25.5)	1,836 (21.0)	0.0004
History of stroke	415 (35.8)	1,326 (15.1)	<0.001
History of CAD	192 (16.6)	338 (3.9)	<0.001
Lab, mean(SD)			
Systolic blood pressure, mmHg	149.0 (27.1)	148.9 (27.5)	0.88
Diastolic blood pressure, mmHg	84.3 (15.3)	87.0 (15.9)	<0.001
Admission glucose, mg/dL	126.2 (53.1)	123.2 (51.6)	0.075
Total cholesterol, mg/dL	162.1 (41.9)	184.8 (40.9)	<0.001
LDL cholesterol, mg/dL	92.1 (33.7)	113.2 (35.6)	<0.001
Stroke characteristics			
Median onset to arrival time (IQR), hours	5.0 (1.5–16.0)	5.7 (2.0–16.8)	0.004
Mean initial NIHSS score (95%CI)	5.9 (5.5–6.3)	6.5 (6.3–6.6)	<0.001
Median initial NIHSS score (IQR)	5 (2–8)	4 (2–9)	<0.001
Stroke subtype, *n* (%)			<0.001
LAA	417 (36.0)	3,099 (35.4)	
SVO	176 (15.2)	1,879 (21.5)	
CE	325 (28.0)	2,038 (23.3)	
UDE or ODE	242 (20.9)	1,740 (19.9)	
Discharge mRS 0 to 2	738 (63.6)	4,970 (56.8)	<0.001
Pre-stroke medication, *n* (%)			
Any antiplatelet	803 (69.2)	1,780 (20.3)	<0.001
ARB+ACEI	520 (44.8)	1,424 (16.3)	<0.001
Beta-blocker	267 (23.0)	609 (7.0)	<0.001
Diuretic	219 (18.9)	667 (7.6)	<0.001
Calcium channel blocker	397 (34.2)	1,335 (15.3)	<0.001

* P-values are calculated by Student's t-test, Pearson chi-square test, or Wilcoxon rank sum test as appropriate

mRS, modified Rankin scale; CAD, coronary artery disease; ARB, angiotensin-receptor blocker; ACEI, angiotensin converting enzyme inhibitor; NIHSS, National Institutes of Health Stroke Scale; SD, standard deviation; IQR, interquartile range; LAA, large-artery atherosclerosis; SVO,

small-vessel occlusion; CE, cardioembolism; UDE, stroke of undetermined etiology; ODE, stroke of other determined etiologies

Table S4. Odd ratios for dichotomized NIHSS score by prestrroke statin use (*n*=8,340)

	OR	95% CI	p-value
Before PS			
Unadjusted	1.38	(1.20 –1.60)	<0.001
Adjusted*	1.25	(1.03 –1.50)	0.021
After PS			
PS-matched[‡]	1.16	(0.95 –1.43)	0.149
PS-stratification, deciles[¶]	1.18	(0.98 –1.44)	0.088

OR for mild stroke defined as NIHSS score 0 to 4
* Adjusted for age, sex, BMI, DBP, Hemoglobin, total cholesterol, LDL cholesterol, previous mRS, history of hypertension, diabetes mellitus, hyperlipidemia, atrial fibrillation, stroke, CAD, smoking, prior medication of any antiplatelet, Anticoagulant, ARB+ACEI, Beta-blocker, Diuretics, Calcium channel blocker, TOAST classification, OTA, and SYSO.
Matched on PS = 618 pairs (2203, 1:1 matching = 225 pairs, 1:2 matching = 80 pairs, 1:3 matching = 52 pairs, 1:4 matching = 261 pairs)
† Adjusted for hyperlipidemia, history of stroke, total cholesterol, LDL cholesterol, prior medications of any antiplatelet and ARB or ACEI, and SYSO
¶ Adjusted for SYSO

Table S5. Comparison of the initial NIHSS Scores between statin users and non-users in patients not treated with thrombolytic therapy (*n*=8,340)

	Unadjusted				Adjusted*			
	Statin users	Non-users	Difference between means	P-value	Statin users	Non-users	Difference between means	P-value†
All patients, *n*	964	7,376			964	7,376		
Mean (95% CI)	4.6 (4.3 - 4.9)	5.4 (5.3 - 5.6)	0.8 (0.5 - 1.2)	<0.001	5.6 (5.0 - 6.1)	6.2 (5.7 - 6.7)	0.6 (0.2 - 1.1)	0.005
Median (IQR)	3.0 (1.0 - 6.0)	3.0 (2.0 - 7.0)		<0.001				
LAA, *n*	361	2,678			361	2,678		
Mean (95% CI)	4.0 (3.6 - 4.5)	5.5 (5.3 - 5.7)	1.4 (1.0 - 1.9)	<0.001	9.1 (6.6 - 11.5)	9.8 (7.4 - 12.3)	0.8 (0.1 - 1.5)	0.031
Median (IQR)	3.0 (1.0 - 5.0)	4.0 (2.0 - 7.0)		<0.001				
SVO, *n*	168	1,790			168	1,790		
Mean (95% CI)	2.5 (2.2 - 2.8)	3.1 (3.0 - 3.2)	0.6 (0.3 - 0.93)	<0.001	2.8 (0.5 - 5.1)	3.3 (1.0 - 5.6)	0.5 (0.1 - 0.9)	0.029
Median (IQR)	2.0 (1.0 - 4.0)	3.0 (1.0 - 4.0)		<0.001				
CE, *n*	234	1,477			234	1,477		
Mean (95% CI)	7.6 (6.7 - 8.5)	8.3 (8.0 - 8.7)	0.7 (-0.3 - 1.7)	0.155	7.9 (6.6 -9.1)	7.9 (6.7 - 9.0)	-0.0 (-1.2 - 1.2)	0.99
Median (IQR)	5.0 (2.0 - 12.0)	6.0 (2.0 - 14.0)		0.131				
UDE or ODE, *n*	201	1,431			201	1,431		
Mean (95% CI)	4.0 (3.3 - 4.6)	5.4 (5.1 - 5.7)	1.4 (0.7 - 2.2)	<0.001	5.7 (4.5 - 7.0)	6.6 (5.5 - 7.8)	0.9 (-0.6 – 2.0)	0.097
Median (IQR)	3.0 (1.0 - 5.0)	3.0 (1.0 - 7.0)		0.002				

Values are mean (95% confidence interval) for unadjusted analyses and least-square mean (95% confidence interval) for adjusted analyses.

*Adjusted variables are age, sex, body mass index,diastolic blood pressure, hemoglobin, total cholesterol, LDL cholesterol, previous mRS, history of hypertension, diabetes mellitus, hyperlipidemia, atrial fibrillation, history of stroke, history of coronary artery disease, smoking, previous medications, onset to arrival time, and TOAST classification.

†P-value obtained using analysis of covariance.

NIHSS, National Institutes of Health Stroke Scale; LAA, large-artery atherosclerosis; SVO, small-vessel occlusion; CE, cardioembolism; UDE, stroke of undetermined etiology; ODE, stroke of other determined etiologies

Table S6. Comparison of the initial NIHSS Scores between statin users and non-users for all patients including non-thrombolysed and thrombolysed patients (*n*=9,916)

	Unadjusted				Adjusted*			
	Statin users	Non-users	Difference between means	P-value	Statin users	Non-users	Difference between means	P-value†
All patients, *n*	1160	8756			1,160	8,756		
Mean (95% CI)	5.9 (5.5 - 6.3)	6.5 (6.3 - 6.6)	0.6 (0.2 - 1.0)	0.003	6.8 (6.2 - 7.3)	7.2 (6.7 - 7.7)	0.5 (0.0 - 0.9)	0.038
Median (IQR)	3.0 (2.0 - 8.0)	4.0 (2.0 - 9.0)		<0.001				
LAA, *n*	417	3099			417	3,099		
Mean (95% CI)	4.9 (4.4 - 5.3)	6.2 (6.0 - 6.4)	1.4 (0.8 - 1.9)	<0.001	9.9 (7.4 - 12.3)	10.6 (8.2 - 13.0)	0.8 (0.0 - 1.5)	0.039
Median (IQR)	3.0 (2.0 - 6.0)	4.0 (2.0 - 9.0)		<0.001				
SVO, *n*	176	1879			176	1,879		
Mean (95% CI)	2.7 (2.4 - 3.1)	3.2 (3.1 - 3.3)	0.5 (0.1 - 0.9)	0.014	3.0 (0.5 - 5.4)	3.3 (0.8 - 5.8)	0.3 (-0.1 - 0.8)	0.147
Median (IQR)	2.0 (1.0 - 4.0)	3.0 (1.0 - 4.0)		0.002				
CE, *n*	325	2038			325	2,038		
Mean (95% CI)	9.5 (8.7 - 10.4)	9.8 (9.5 - 10.2)	0.3 (-0.6 - 1.2)	0.48	9.6 (8.5 - 10.7)	9.3 (8.3 - 10.3)	-0.3 (-1.4 - 0.7)	0.52
Median (IQR)	7.0 (3.0 - 16.0)	9.0 (3.0 - 16.0)		0.33				
UDE or ODE, *n*	242	1740			242	1,740		
Mean (95% CI)	5.1 (4.4 - 5.8)	6.5 (6.2 - 6.8)	1.4 (0.6 - 2.2)	<0.001	6.7 (5.5 - 8.0)	7.5 (6.3 - 8.7)	0.8 (-0.3 - 1.8)	0.146
Median (IQR)	3.0 (1.0 - 5.0)	4.0 (2.0 - 9.0)		0.002				

Values are mean (95% confidence interval) for unadjusted analyses and least-square mean (95% confidence interval) for adjusted analyses.

*Adjusted variables are age, sex, body mass index, diastolic blood pressure, hemoglobin, total cholesterol, LDL cholesterol, previous mRS, history of hypertension, diabetes mellitus, hyperlipidemia, atrial fibrillation, history of stroke, history of coronary artery disease, smoking, previous medications, onset to arrival time, and TOAST classification.

†P-value obtained using analysis of covariance.

NIHSS, National Institutes of Health Stroke Scale; LAA, large-artery atherosclerosis; SVO, small-vessel occlusion; CE, cardioembolism; UDE, stroke of undetermined etiology; ODE, stroke of other determined etiologies

Table S7. Comparisons of discharge mRS outcome by statin use during hospitalization in pre-stroke statin non-users.

	Binary outcome*		Ordinal outcome†	
	OR (95% CI)	P-value	OR (95% CI)	P-value
Crude analysis	1.35 (1.22–1.50)	<.001	1.49 (1.36–1.63)	<.001
Multivariable analysis‡	1.08 (0.94–1.24)	0.309	1.26 (1.14–1.40)	<.001

Odd ratio for pre-stroke statin (-) & statin use during hospitalization (n=5,428) vs. pre-stroke statin (-) & no statin use during hospitalization (n=1,892)

* Dependent variable: mRS 0 to 2 versus 3 to 6

†Dependent variable: six mRS levels by collapsing mRS 5 and mRS 6 into a single level

‡ Adjusted for age, sex, body mass index, diastolic blood pressure, hemoglobin, total cholesterol, LDL cholesterol, pre-stroke modified Rankin scale score, history of hypertension, diabetes mellitus, hyperlipidemia, atrial fibrillation, history of stroke, history of coronary artery disease, smoking, prior medication of any antiplatelet, anticoagulant, angiotensin receptor blocker or angiotensin converting enzyme inhibitor, beta-blocker, diuretics, calcium-channel blocker, TOAST classification, onset to arrival time, and initial NIHSS score

Table S8. Adjusted odds ratios of achieving a mRS 0-2 outcome and favorable shifting of the mRS score among pre-stroke statin users stratified by TOAST subtypes (*n*=8,340)

TOAST classification	mRS 0-2 dichotomization		Ordinal analysis	
	OR (95% CI)	P-value	OR (95% CI)	P-value
Large artery atherosclerosis	1.37 (0.99 –1.91)	0.061	1.42 (1.11–1.81)	0.006
Small vessel occlusion	1.42 (0.84–2.40)	0.194	1.08 (0.77–1.54)	0.65
Cardioembolism	1.74 (1.09–2.76)	0.019	1.23 (0.89–1.70)	0.22
Others	1.68 (1.01–2.79)	0.045	1.13 (0.79–1.60)	0.50

Adjusted for age, sex, body mass index, diastolic blood pressure, total cholesterol, LDL cholesterol, pre-stroke mRS, hypertension, diabetes mellitus, hyperlipidemia, atrial fibrillation, history of prior stroke and coronary heart disease, smoking, prior use of antiplatelet, ARB or ACEI, beta-blocker, diuretics, and CCB, initial NIHSS score, and onset-to-arrival time

Table S9. Multivariable analysis for favorable mRS outcome for all patients (*n*=9,916)

	Binary logistic regression*			Ordinal logistic regression†		
	OR	95% CI	P-value	OR	95% CI	P-value
Demographic						
Age, years	0.98	(0.97–0.98)	<0.001	0.98	(0.98–0.99)	<0.001
Sex, male	1.24	(1.09–1.41)	<0.001	1.12	(1.02–1.23)	0.019
Pre-stroke mRS						
1	Ref			Ref		
0	1.31	(1.08–1.59)	0.007	1.51	(1.30–1.75)	<0.001
BMI	1.01	(0.99–1.02)	0.25	1.01	(1.00–1.02)	0.27
Risk factors						
Hypertension	1.03	(0.91–1.15)	0.66	1.00	(0.92–1.09)	0.96
DM	0.74	(0.67–0.82)	<0.001	0.75	(0.69–0.81)	<0.001
Hyperlipidemia	0.86	(0.77–0.98)	0.019	0.92	(0.84–1.01)	0.072
Smoking	0.94	(0.83–1.06)	0.30	1.02	(0.93–1.11)	0.70
Atrial fibrillation	1.07	(0.87–1.30)	0.53	0.91	(0.79–1.06)	0.23
History of stroke	0.95	(0.82–1.09)	0.44	0.93	(0.84–1.04)	0.20
History of CAD	0.83	(0.66–1.04)	0.099	0.89	(0.75–1.05)	0.155
Lab						
DBP, (unit 10 mmHg)	0.96	(0.93–0.99)	0.008	0.95	(0.93–0.97)	<0.001
Hemoglobin	1.03	(1.00–1.06)	0.074	1.03	(1.00–1.05)	0.033
Total cholesterol, (unit 10 mg/ dL)	0.95	(0.93–0.97)	<0.001	0.96	(0.94–0.98)	<0.001
LDL cholesterol, (unit 10 mg/ dL)	1.04	(1.01–1.07)	0.004	1.03	(1.01–1.05)	0.014
Stroke characteristics						
Initial NIHSS	0.80	(0.79–0.81)	<0.001	0.79	(0.79–0.80)	<0.001
Onset to arrival time	0.99	(0.99–1.00)	<0.001	0.99	(0.99–0.99)	<0.001
Stroke subtype						
LAA	Ref			Ref		
SVO	1.61	(1.41–1.84)	<0.001	1.27	(1.15–1.40)	<0.001
CE	1.34	(1.09–1.63)	0.004	1.27	(1.10–1.48)	0.001
UDE or ODE	1.26	(1.10–1.45)	<0.001	1.18	(1.06–1.30)	0.002
Pre-stroke medication						
Antiplatelet	1.02	(0.90–1.16)	0.76	1.00	(0.91–1.10)	1.00
Anticoagulant	0.99	(0.77–1.29)	0.96	1.03	(0.85–1.25)	0.76
ARB or ACEI	1.11	(0.97–1.27)	0.140	1.03	(0.93–1.15)	0.52
Beta-blocker	1.32	(1.09–1.59)	0.005	1.17	(1.02–1.35)	0.024
Diuretics	1.04	(0.86–1.25)	0.71	1.00	(0.87–1.14)	0.97
Calcium channel blocker	1.00	(0.87–1.14)	0.95	1.08	(0.97–1.19)	0.170
Statin use during hospitalization	1.27	(1.13–1.43)	<0.001	1.49	(1.36–1.62)	<0.001
Pre-stroke statin use	1.44	(1.20–1.74)	<0.001	1.22	(1.06–1.40)	0.006

* Dependent variables: mRS 0 to 2 versus 3 to 6
†Dependent variables: six levels by collapsing mRS 5 and mRS 6 into a single level
mRS, modified Rankin scale; CAD, coronary artery disease; ARB, angiotensin-receptor blocker; ACEI, angiotensin converting enzyme inhibitor; NIHSS, National Institutes of Health Stroke

Scale; SD, standard deviation; IQR, interquartile range; LAA, large-artery atherosclerosis; SVO, small-vessel occlusion; CE, cardioembolism; UDE, stroke of undetermined etiology; ODE, stroke of other determined etiologies

Table S10. Comparisons of initial NIHSS scores between statin users and non-users by median onset to arrival time.

Initial NIHSS	Onset to arrival < 7.6h							Onset to arrival ≥ 7.6h							P-value†
	Statin users		Non-users		Difference between means		P-value	Statin users		Non-users		Difference between means		P-value	
Before PS															
Unadjusted	5.2	(4.7-5.7)	6.4	(6.2-6.6)	1.21	(0.69-1.73)	<0.001	4.0	(3.5-4.5)	4.5	(4.3-4.7)	0.53	(-0.02-1.07)	0.0581	0.0762
Adjusted*	5.7	(5.1-6.4)	6.7	(6.2-7.3)	1.00	(0.47-1.54)	0.0002	5.7	(5.1-6.4)	6.0	(5.5-6.5)	0.29	(-0.26-0.85)	0.2981	0.0429

Values are Mean (95% CI) or Least-square mean (95% CI) as appropriate.

* Adjusted for age, sex, body mass index, diastolic blood pressure, hemoglobin, total cholesterol, LDL cholesterol, pre-stroke mRS, hypertension, diabetes mellitus, hyperlipidemia, atrial fibrillation, history of prior stroke and coronary heart disease, smoking, prior use of antiplatelet, ARB or ACEI, beta-blocker, diuretics, CCB, TOAST classification, and SYSO

† P-value by interaction effect between statin use prior and onset to arrival

Table S11. Comparisons of discharge mRS outcomes by median onset to arrival time.

Outcomes		Onset to arrival < 7.6h		Onset to arrival ≥ 7.6h		P-value
		OR (95% CI)	P-value	OR (95% CI)	P-value	
Dichotomized mRS	Unadjusted	1.51 (1.24–1.84)	<0.001	1.38 (1.12–1.70)	0.003	0.53
	Adjusted	1.57 (1.19–2.08)	0.002	1.53 (1.16–2.01)	0.002	0.87
Ordinal mRS	Unadjusted	1.55 (1.31–1.82)	<0.001	1.23 (1.03–1.46)	0.020	0.056
	Adjusted	1.40 (1.15–1.70)	0.001	1.20 (0.98–1.46)	0.074	0.22

OR for stain use prior to stroke

* Adjusted for age, sex, body mass index, diastolic blood pressure, hemoglobin, total cholesterol, LDL cholesterol, pre-stroke mRS, hypertension, diabetes mellitus, hyperlipidemia, atrial fibrillation, history of prior stroke and coronary heart disease, smoking, prior use of antiplatelet, ARB or ACEI, beta-blocker, diuretics, CCB, TOAST classification, and SYSO

† P-value by interaction effect between statin use prior and onset to arrival

Figure S1. Study flow diagram

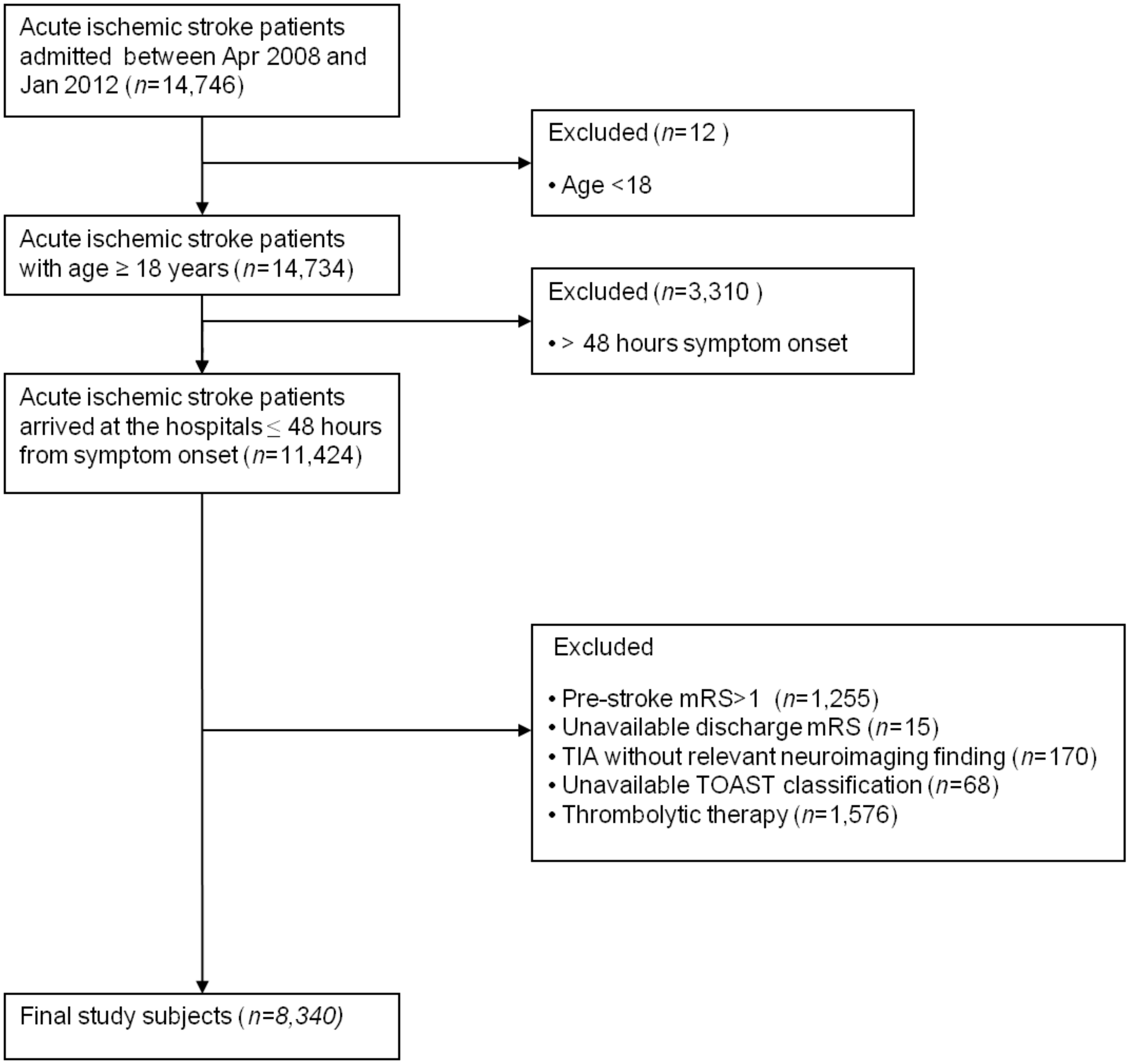

mRS, modified Rankin scale; TOAST, Trial of Org 10172 in Acute Stroke Treatment

Figure S2. Standardized difference of covariates before and after propensity score matching.

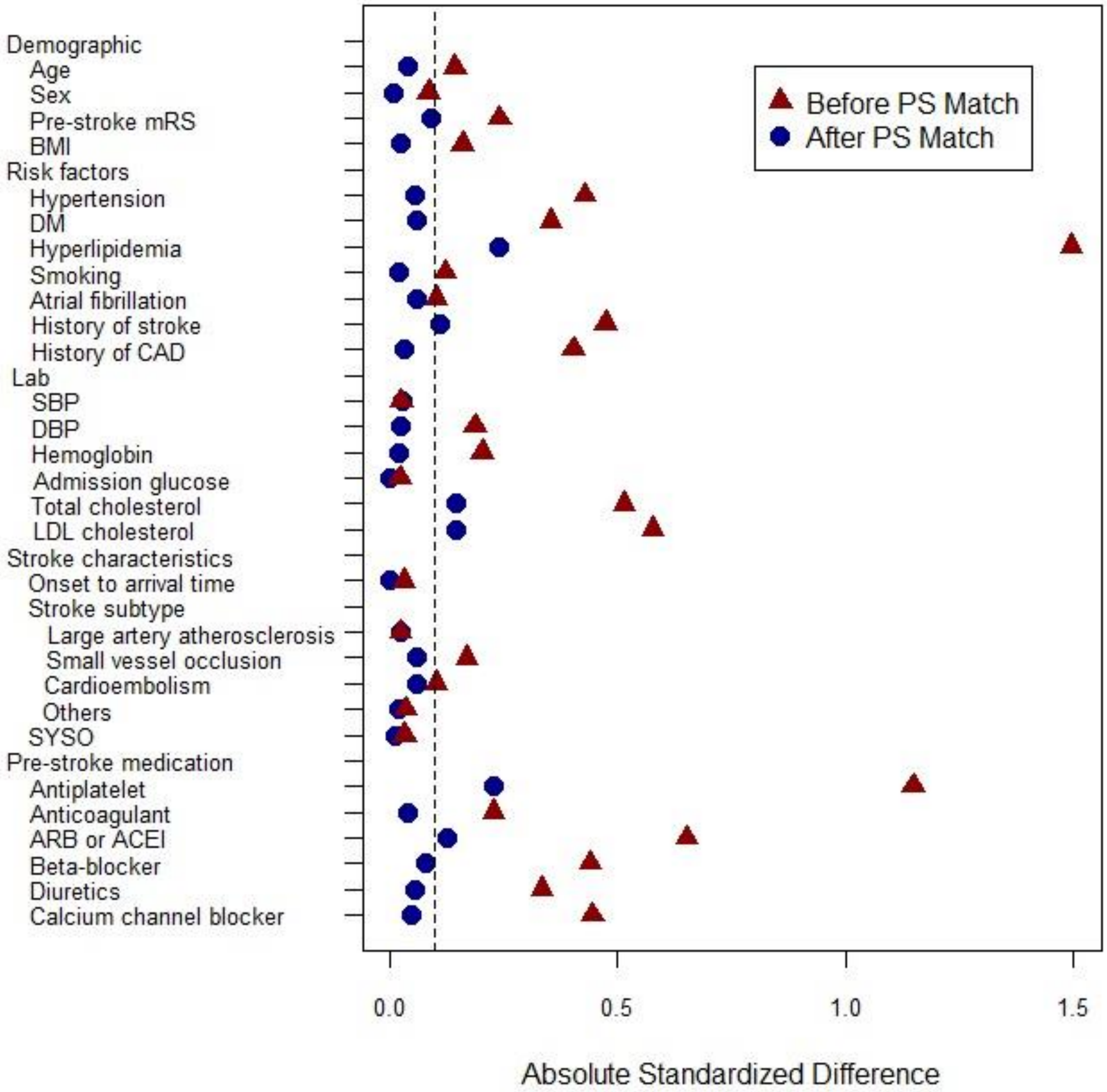

PS, Propensity score; BMI, body mass index; mRS, modified Rankin scale; DM, diabetes mellitus; CAD, coronary artery disease; SBP, systolic blood pressure; DBP, diastolic blood pressure; ARB, angiotensin-receptor blocker; ACEI, angiotensin converting enzyme inhibitor

Figure S3. Comparison of the initial NIHSS scores by ischemic stroke subtype in 8,340 patients

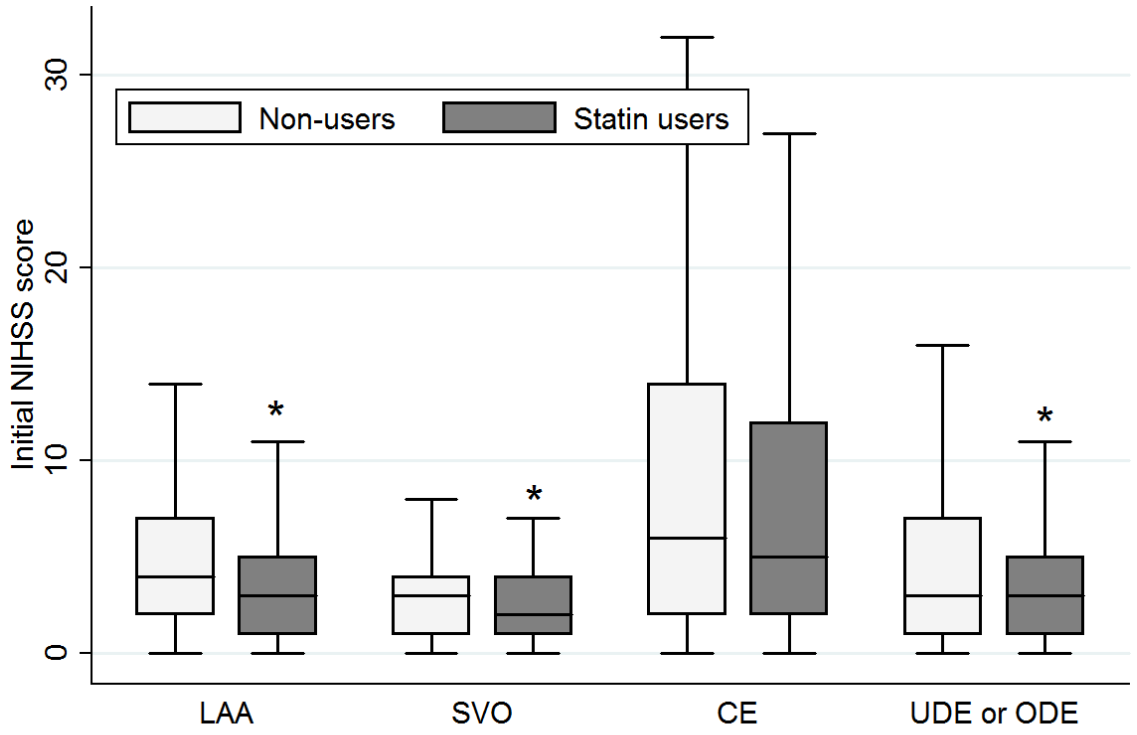

NIHSS, National Institutes of Health Stroke Scale; LAA, large-artery atherosclerosis; SVO, small-vessel occlusion; CE, cardioembolism; UDE, stroke of undetermined etiology; ODE, stroke of other determined etiologies

* p<0.05 on t-test and Wilcoxon rank-sum test
